# Association of Abnormal Cardiac Biomarkers and Cardiovascular Complications, with Mortality in Patients with SARS-CoV-2 Infection in Latin America

**DOI:** 10.3390/jcdd11070205

**Published:** 2024-06-30

**Authors:** Juan Esteban Gómez-Mesa, Manuela Escalante, Juan Andrés Muñoz-Ordoñez, Valeria Azcárate-Rodriguez, Juan David Peláez-Martínez, Andrea Alejandra Arteaga-Tobar, Hoover León-Giraldo, Andrea Valencia-Orozco, Eduardo Roque Perna, Alexander Romero, Iván Mendoza, Fernando Wyss, José Luis Barisani, Mario Speranza, Walter Alarco, Cesar Herrera, Julián Lugo-Peña, Liliana Patricia Cárdenas-Aldaz, Victor Rossel, Daniel Sierra

**Affiliations:** 1 Departamento de Cardiología, Fundación Valle del Lili, Cali 760032, Colombia; andrea.arteaga@fvl.org.co; 2 Facultad de Ciencias de Salud, Universidad Icesi, Cali 760031, Colombia; manuela.escalante@fvl.org.co (M.E.); juan.munoz.or@fvl.org.co (J.A.M.-O.); valeria.azcarate@fvl.org.co (V.A.-R.); jdpm0531@gmail.com (J.D.P.-M.); 3 Centro de Investigaciones Clínicas, Fundación Valle del Lili, Cali 760032, Colombia; hoover.leon.gi@fvl.org.co (H.L.-G.); andrea.valencia.or@fvl.org.co (A.V.-O.); 4 Departamento de Cardiología, Instituto de Cardiología JF Cabral, Corrientes 3400, Argentina; pernaucic@hotmail.com; 5 Departamento de Cardiología, Hospital Santo Tomas, Panama City 07093, Panama; alexrom2876@me.com; 6 Departamento de Cardiología, Universidad Central de Venezuela, Caracas 1040A, Venezuela; imivanjm@gmail.com; 7 Departamento de Cardiología, Servicios y Tecnología Cardiovascular de Guatemala S.A–Cardiosolutions, Guatemala City 01010, Guatemala; fernandowyss@gmail.com; 8 Departamento de Cardiología, Clínica Adventista Belgrano, Buenos Aires 1710, Argentina; jlbarisani@gmail.com; 9 Departamento de Cardiología, Hospital Clínica Bíblica, San Jose 10104, Costa Rica; sacagce@icloud.com; 10 Departamento de Cardiología, Instituto Nacional Cardiovascular INCOR ESSALUD, Lima 15072, Peru; walarco@hotmail.com; 11 Departamento de Cardiología, Centro de Diagnóstico, Medicina Avanzada y Telemedicina (CEDIMAT), Santo Domingo 10216, Dominican Republic; cjherrera@cedimat.net; 12 Departamento de Cardiología, Clínica del Occidente, Bogota 110110, Colombia; jrlugop@gmail.com; 13 Departamento de Cardiología, Hospital Eugenio Espejo, Quito 170136, Ecuador; lilicarld@gmail.com; 14 Departamento de Cardiología, Hospital del Salvador, San Salvador 1101, El Salvador; vrosselm@yahoo.es; 15 Departamento de Cardiología, Instituto Nacional de Cardiología–Ignacio Chávez, Mexico City 14080, Mexico; danielsierralaram@gmail.com

**Keywords:** COVID-19, SARS-CoV-2, biomarkers, cardiovascular disease, mortality, prognosis

## Abstract

Background: The COVID-19 pandemic has highlighted a correlation between cardiac complications and elevated cardiac biomarkers, which are linked to poorer clinical outcomes. Objective: This study aims to determine the clinical impact of cardiac biomarkers in COVID-19 patients in Latin America. Subjects and methods: The CARDIO COVID 19-20 Registry is a multicenter observational study across 44 hospitals in Latin America and the Caribbean. It included hospitalized COVID-19 patients (*n* = 476) who underwent troponin, natriuretic peptide, and D-dimer tests. Patients were grouped based on the number of positive biomarkers. Results: Among the 476 patients tested, 139 had one positive biomarker (Group C), 190 had two (Group B), 118 had three (Group A), and 29 had none (Group D). A directly proportional relationship was observed between the number of positive biomarkers and the incidence of decompensated heart failure. Similarly, there was a proportional relationship between the number of positive biomarkers and increased mortality. In Group B, patients with elevated troponin and natriuretic peptide and those with elevated troponin and D-dimer had 1.4 and 1.5 times higher mortality, respectively, than those with elevated natriuretic peptide and D-dimer. Conclusions: In Latin American COVID-19 patients, a higher number of positive cardiac biomarkers is associated with increased cardiovascular complications and mortality. These findings suggest that cardiac biomarkers should be utilized to guide acute-phase treatment strategies.

## 1. Introduction

The SARS-CoV-2 virus is a β-coronavirus belonging to the Coronaviridae family, formed by a single positive-sense RNA strand. It causes the severe acute respiratory syndrome known as COVID-19, a global epidemic disease responsible for more than 7.04 million as of May 2024, according to WHO estimates [1,2,3,4,5]. While COVID-19 primarily affects the respiratory system, current evidence suggests significant impacts on the cardiovascular system as well, leading to various cardiac manifestations, including chest pain, palpitations, myocardial injury, and exacerbation of underlying cardiovascular diseases [6,7]. Myocardial injury has been reported in up to 27.8% of hospitalized COVID-19 patients in China [8].

Patients with COVID-19 and underlying cardiovascular diseases generally present with more severe symptoms and a higher mortality rate compared to those with other comorbidities, such as diabetes or chronic respiratory diseases [9,10,11,12,13,14,15]. Cardiac biomarkers are proposed to play a critical role in the diagnosis, prognosis, and management of COVID-19 patients. Robust evidence links elevated biomarkers of myocardial damage and stress, such as cardiac troponin and natriuretic peptides, with increased morbidity and mortality in these patients [9,11,16,17].

Among cardiac biomarkers, troponin is the most strongly associated with adverse outcomes and mortality during COVID-19, with an almost universal increase observed in critically ill or deceased patients. The one-month mortality rate in patients with elevated troponin levels exceeds 50% [8,18,19]. Natriuretic peptides have also been shown to predict COVID-19 severity, with higher values reported in patients who died, suggesting their use as a tool to discriminate severe cases [19,20,21]. Elevated D-dimer levels have been associated with adverse outcomes and increased mortality, although the data is limited and based on small, heterogeneous cohorts. D-dimer values ≥ 1 mg/mL are proposed to have moderate sensitivity and specificity for predicting severe outcomes and fatal events [22].

Cardiovascular diseases are the leading cause of death worldwide, and their prevalence is increasing [23]. This underscores the importance of close monitoring and diagnosis, particularly in the context of COVID-19, which continues to trigger cardiovascular complications and remains a frequent cause of consultation [24].

Given this context, we aim to describe the relationship between cardiac injury biomarkers and the presence of cardiovascular complications and in-hospital mortality in patients hospitalized due to COVID-19 in Latin America.

## 2. Subjects and Methods

### 2.1. Supervision and Data Collection

This study was observational, analytical, retrospective, and multicenter, focusing on patients hospitalized due to confirmed SARS-CoV-2 infection. The included subjects were part of the CARDIO COVID 19-20 registry (Latin American Registry of Cardiovascular Diseases and COVID-19), established by the Inter-American Heart Failure Council (CIFACAH) of the Inter-American Society of Cardiology (SIAC).

The study population consisted of adult patients aged 18 years or older who met the following inclusion criteria: serologically confirmed diagnosis of SARS-CoV-2 infection according to the WHO definition, hospitalization for more than 24 h for the treatment of COVID-19, and available results for troponin, natriuretic peptide, and d-dimer. Patients without complete clinical histories were excluded. Follow-up for recruited patients occurred 30 days after hospital discharge. Both patients with and without previous cardiovascular comorbidities reported in their clinical history were included.

The registry was designed, developed, and conducted by CIFACAH of the SIAC, and coordinated by the Clinical Research Center (CRC) of the Fundación Valle de Lili (FVL) in Cali, Colombia. The study complied with the Declaration of Helsinki and did not require informed consent as no additional interventions were performed. The protocol was approved by the CRC and the Human Ethics Committee of FVL (1835), as well as the Executive Committee of CIFACAH/SIAC. The data were obtained from medical records and collected using the REDCap (Research Electronic Data Capture) electronic database system. REDCap 14.3.13—© 2024 Vanderbilt University

### 2.2. Participants

**Figure 1:**
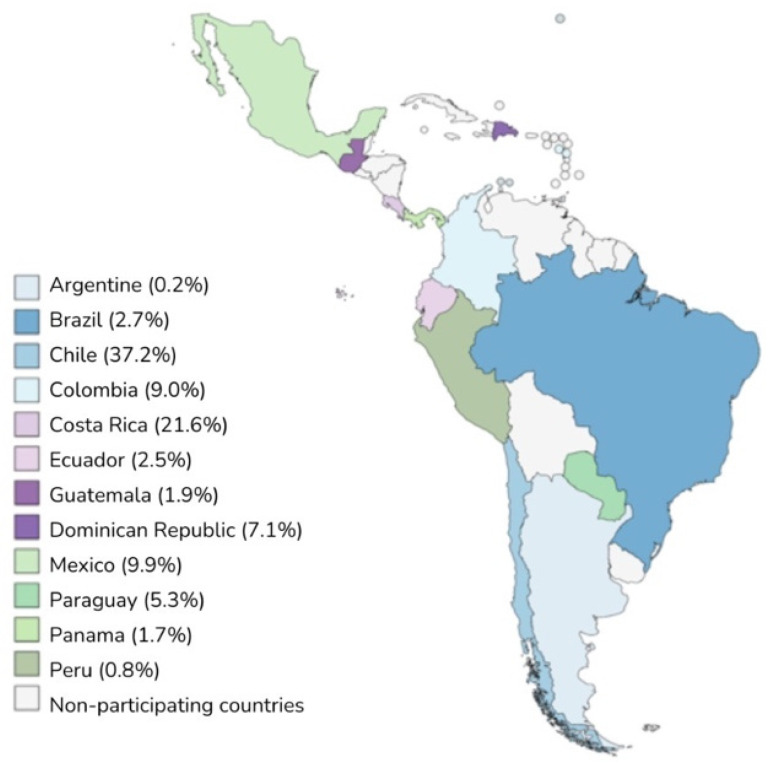
Distribution of patients in the participating countries.

The CARDIO COVID 19-20 registry included 3260 patients admitted to hospitalization, emergency, or intensive care units (ICU) in 44 hospitals across 14 Latin American countries between 1 May 2020 and 30 June 2021 (Figure 1). Of these, 476 patients had all three biomarkers measured. 

**Figure 2:**
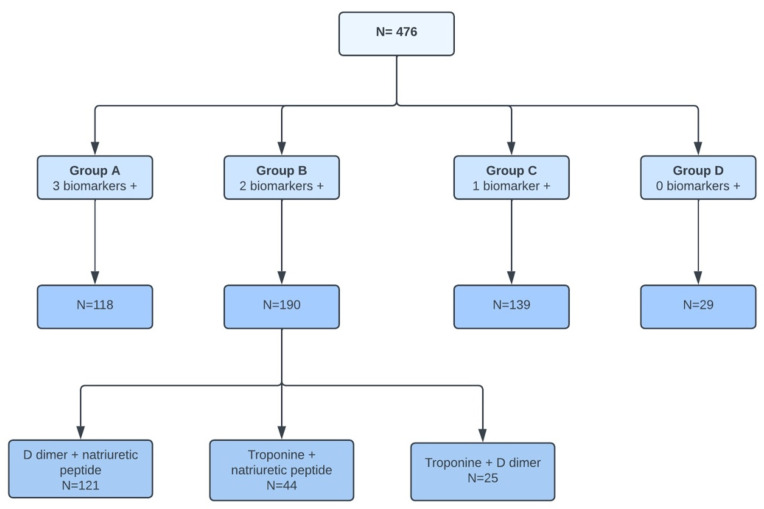
Flowchart of patient distribution.

These patients were divided into four groups (Figure 2) based on the number of abnormal or positive biomarkers:

- Group A: 3 positive biomarkers, with 118 patients
- Group B: 2 positive biomarkers, with 190 patients
- Group C: 1 positive biomarker, with 139 patients
- Group D: No positive biomarkers, with 29 patients

### 2.3. Para-Clinical Tests

The laboratory tests were conducted according to the institutional protocols of each participating center. The measured parameters included inflammatory, metabolic, cardiac, and coagulation markers, among other paraclinical tests. Specifically, cardiac serum biomarkers measured included d-dimer, various types of natriuretic peptides (NP), and different types of troponins. Cardiac biomarkers were grouped by type and classified according to their results as normal/negative or abnormal/positive.

### 2.4. Statistical Analysis

A univariate descriptive analysis was conducted to characterize the behavior of the numerical variables. The normality of the quantitative variables was assessed using the Shapiro–Wilk test. Variables with a *p*-value > 0.05 were considered normally distributed and were presented as means and standard deviations. Variables that did not meet the normality assumption were presented as medians and interquartile ranges. Categorical variables were expressed as proportions, and comparisons between groups were performed using the Chi-square test or Fisher’s exact test, as appropriate. Quantitative variables with a normal distribution were compared using the Student’s *t*-test, while those with a non-normal distribution were compared using the Mann–Whitney test. For paired samples, the Wilcoxon test was employed.

A subclassification of the data obtained in group B (2 positive biomarkers) was performed, depending on the biomarkers that were elevated or abnormal. This subclassification was made with three groups: Troponin + NP, troponin + d-dimer, and NP + d-dimer, with which the comparisons were made according to the condition at discharge.

Subsequently, the association between each of these groups and the outcome of mortality was evaluated. For this comparison, the subgroup with elevated d-dimer plus NP was taken as the reference, considering the group with the lowest mortality and highest prevalence.

All statistical analyses were performed using R V.4.1.1 (R Foundation for Statistical Computing, Vienna, Austria) through R Studio V.1.4.1717.

## 3. Results

### 3.1. Clinical Characteristics at Admission

Of 3260 patients admitted, only 476 were included in various subgroups as given in Table 1. The median age was 64 years, range 52–73 years with a predominance of male gender (68.3% vs. 31.7%). Group A had a median age of 69 years (IQR: 58–77), with a progressive decrease observed across the groups, reaching a median age of 49 years (IQR: 40–69) in group D. The most frequent comorbidities across the four groups were obesity/overweight, which predominated in groups B and C (63.7% and 58.3%, respectively) and were less common in groups A and D (50.8% and 51.7%, respectively) (*p* = 0.14). Hypertension prevalence decreased significantly as the number of positive biomarkers decreased (group A: 66.9%, group B: 60%, group C: 49.6%, and group D: 37.9%; *p* = 0.005). Similarly, dyslipidemia followed the same trend as hypertension, with a statistically significant decrease in prevalence corresponding to a lower number of positive biomarkers (group A: 23.7%, group B: 20.5%, group C: 7.2%, and group D: 6.9%; *p* < 0.001) (Table 1).

The most frequent clinical manifestation at admission was dyspnea, with a similar prevalence across all groups (79.7%, 83.2%, 75.5%, and 69%, respectively; *p* = 0.2). No significant differences were observed in vital signs, except for arterial oxygen saturation. The median arterial oxygen saturation values were: group A: 90.5% (IQR: 83–96%), group B: 90% (IQR: 84–94%), group C: 90% (IQR: 86–94%), and group D: 94% (IQR: 92–96%). This indicates that group D had significantly higher saturation compared to the other groups (*p* = 0.013).

### 3.2. Paraclinical Tests at Admission

**Figure 3:**
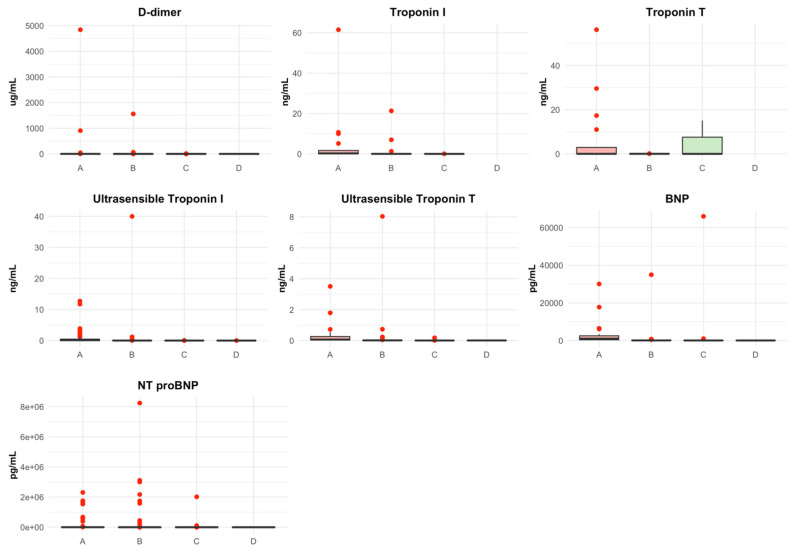
Box plot of Biomarkers value distribution. Abnormal or positive biomarker value: D-dimer, >0.5 ug/mL, Troponin I high sensitivity (men), >0.0342 ng/mL, Troponin I high sensitivity (women), >0.0156 ng/mL, Troponin T high sensitivity, >0.014 ng/mL, Troponin T, >0.1 ng/mL, Troponin I (men), >0.033 ng/mL, Troponin I (women), >0.013 ng/mL, BNP, >266 pg/mL, NT-proBNP, >125 pg/mL. BNP: B-type natriuretic peptide; NT-proBNP: N-terminal pro-brain natriuretic peptide; Pg: Picogram; Ng: Nanogram; Ug: Microgram; mL: milliliters.

The cardiac biomarkers exhibited a similar trend across the groups, with higher levels observed in patients with more positive or abnormal markers. D-dimer levels were highest in group A (median: 1.54) and decreased progressively in groups C and D (median: 0.30 and 0.28, respectively). Similarly, BNP and NT-proBNP levels decreased as the groups progressed. Troponin I, Troponin T, high-sensitivity Troponin I, and high-sensitivity Troponin T also followed this trend, showing lower values in groups with fewer or no abnormal biomarkers. The distribution of biomarker values is represented in Figure 3.

### 3.3. Imaging Studies during Hospital Stay

On chest X-ray, pulmonary congestion was observed in 36.6% of patients in group A, decreasing significantly as the number of positive biomarkers decreased, reaching 0.0% in group D (*p* < 0.001). Echocardiographic abnormalities, including decreased systolic function, right ventricular dysfunction, pericardial effusion, and valvular dysfunction, were predominantly found in group A, with their incidence progressively decreasing across the groups, reaching the lowest levels in group D (Table 2).

### 3.4. Cardiovascular Complications during Hospitalization

The most frequent cardiovascular complications were acute decompensated heart failure (ADHF) (*p* < 0.001) and cardiac arrhythmias (CA) (*p* = 0.004). ADHF occurred in 17.6% of patients, primarily in group A (35.6%), followed by group B (8.4%) and group C (5%) (*p* < 0.001). Among the ADHF clinical profiles, congestion was the most prevalent (10.9%), followed by cardiogenic shock (4.2%) and pulmonary edema (2.1%). Conversely, CA occurred in 13% of patients, with supraventricular arrhythmias being the most prevalent type at 7.8%, mainly present in group A (22.9%), with fewer events occurring in groups C and D (Table 3).

### 3.5. Treatment for COVID-19

The hospital treatment for COVID-19 provided to patients was similar across the four groups, with notable use of oral and parenteral corticosteroids, chloroquine, lopinavir, and ritonavir. However, anticoagulation therapy was significantly more prevalent in group A and decreased progressively across the groups (group A: 61.9%, group B: 47.9%, group C: 52.5%, group D: 27.6%; *p* = 0.005). Additionally, the use of azithromycin and hydroxychloroquine was highest in group C, with a statistically significant difference compared to the other groups (*p* < 0.001).

### 3.6. Respiratory and Cardiovascular Support

Invasive mechanical ventilation (IMV) was the primary intervention performed, applied to 50.4% of patients. The prevalence of IMV was similar among groups A, B, and C (53.4%, 52.1%, and 51.1%, respectively), but significantly lower in group D (24.1%; *p* = 0.034). Inotropes were used in 17.4% of patients, with a predominance in group A (28.8%) and a progressive decrease to group D in a statistically significant manner (0.0%, *p* < 0.001). On the other hand, vasodilators were used in 6.3% of participants, predominating in group A (11.9%) and decreasing progressively in a significant manner until reaching group D (0.0%, *p* = 0.024) (Table 4).

### 3.7. Outcomes during Hospitalization

Admission to the ICU was numerically more prevalent in groups A and C (73.7% and 74.1%, respectively) compared to groups B and D (67.9% and 55.2%, respectively), but without statistical significance (*p* = 0.15). Mortality was an event exclusive and statistically significant to the groups with positive biomarkers, unlike group D, and was considerably higher in group A (group A = 50%, group B = 31.1%, group C = 18%, and group D = 0%, *p* < 0.001). Additionally, it was observed that patients in group A had a higher prevalence of cardiovascular death (37.3%) compared to groups B and C (28.8%, 12%), (*p* = 0.066). (Table 5)

### 3.8. Outcomes at 30-Day Follow-Up after Hospital Discharge

After 30 days of hospital discharge, no statistically significant differences were observed regarding rehospitalization (*p* = 0.6) or mortality (*p* = 0.2) among the different groups studied (Table 6).

### 3.9. Comparison between Two Abnormal Biomarkers (Group B)

**Figure 4:**
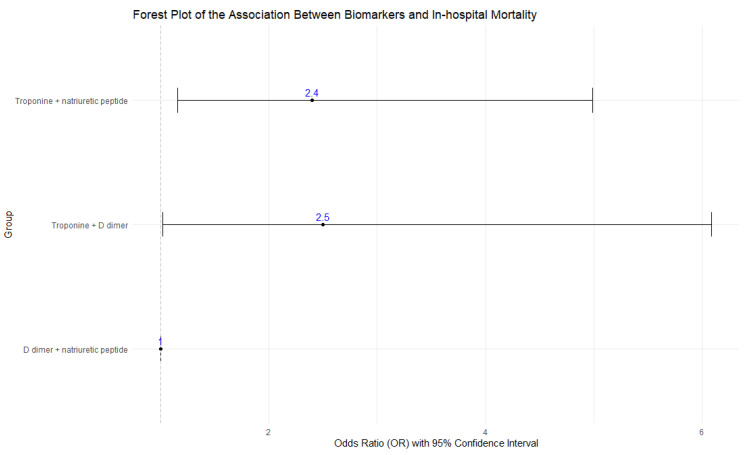
Forest plot of Comparison between two abnormal biomarkers.

When grouping abnormal biomarkers in pairs, the subgroup with elevated D-dimer and NP (used as the reference) had the lowest mortality (24.0%) and the highest prevalence (*n* = 121). The subgroup with elevated troponin and D-dimer reported the highest mortality (44.0%), with a 1.5 times higher risk of death compared to the reference group (OR = 2.5; *p* = 0.045). However, this subgroup was also the least prevalent (*n* = 25). Finally, the group with elevated troponin and NP (*n* = 44) had a mortality rate of 43.2%, with a 1.4 times higher risk of death than the reference group (OR = 2.4; *p* = 0.018). Both associations were statistically significant (Table 7, Figure 4).

## 4. Discussion

### 4.1. General Information

#### 4.1.1. General Characteristics

In general, male patients were more prone to hospitalization secondary to COVID-19, but without statistically significant differences. Conversely, a direct relationship was found between age and the number of positive biomarkers, likely due to a greater number of underlying conditions. Similarly, pathologies associated with metabolic syndrome, such as obesity, hypertension, diabetes, and dyslipidemia, were the most prevalent comorbidities in patients with positive biomarkers. This aligns with findings from other studies conducted in China and the United States, where diabetes, hypertension, and obesity were comorbidities associated with disease severity [25,26,27,28].

#### 4.1.2. Clinical Manifestations

Patients with positive biomarkers presented more symptoms compared to patients with negative or normal biomarkers. However, the group with three positive biomarkers, which corresponds to the group with the highest morbidity and mortality, was not the most symptomatic. Considering that evidence suggests the severity of symptoms is associated with worse clinical outcomes [29], it is proposed that the most severely ill patients may have difficulty expressing their complaints, thus reporting fewer symptoms. It is important to highlight that there is evidence associating dyspnea with a two-fold higher probability of severe disease [30]. However, in our study, there was no significant difference in the frequency of dyspnea across different patient groups.

#### 4.1.3. Paraclinical Tests on Admission

A study conducted at the University Hospital in Wuhan, China, which evaluated various biomarker levels in COVID-19 patients, including Troponin I and NT-proBNP, found that a higher number of positive biomarkers and higher median values were associated with increased severity and mortality [31]. Our study corroborated these findings, showing that group A had higher medians of biomarkers than groups B and C, with group B medians being higher than those of group C. Additionally, a higher median of biomarkers was linked to an increased number of complications and hospital mortality.

#### 4.1.4. In-Hospital Complications

Our study found that patients with positive biomarkers had a higher prevalence of complications during their hospital stay, consistent with previous studies [31,32,33]. Zhu et al.’s meta-analysis reported that elevated biomarkers, including Troponins I and NT-proBNP, were associated with severe COVID-19 forms, similar to Cersosimo et al., who highlighted the role of troponins in predicting mortality [32,33]. The most common complication in our study was acute decompensated heart failure (ADHF), exclusively observed in patients with at least one abnormal biomarker (A = 35.6% vs. D = 0%). These findings are consistent with other studies reporting ADHF as a common complication, associated with more severe disease and worse outcomes [34,35,36,37].

#### 4.1.5. ICU Interventions

Our study showed that the presence of at least one positive cardiovascular biomarker (Troponin, natriuretic peptide, and/or D-dimer) doubled the need for mechanical ventilation compared to patients with negative biomarkers (Table 5). The need for mechanical ventilation increased with the number of positive biomarkers. Furthermore, patients with at least one positive biomarker had a greater need for vasopressor drugs, with the requirement for such drugs increasing with the number of positive biomarkers (Table 5). This is consistent with literature indicating that elevated troponin levels suggest poor prognosis, correlating with a greater need for high-complexity care [38,39].

#### 4.1.6. Anticoagulant Treatment

In this study, a higher need for anticoagulation therapy was observed with an increasing number of positive biomarkers (A = 61.9% vs. D = 27.6%), correlating with the most severe cases of SARS-CoV-2 infection. The literature extensively describes the hematological complications caused by this virus, including thrombotic events [40,41,42,43]. Consequently, a greater need for anticoagulation can be associated with the presence of positive biomarkers and more severe cases [44,45].

#### 4.1.7. Hospital Outcomes

Literature suggests that cardiac biomarkers in COVID-19 patients can predict ICU needs [46,47,48]. However, our study did not find a significant difference in ICU requirements between patients with positive and negative biomarkers. In-hospital mortality was exclusively related to patients with at least one positive biomarker, with a 50% mortality rate in group A and a higher incidence of cardiovascular mortality. Similar findings were reported by Cersosimo et al., indicating that higher levels of Troponin-T and NT-proBNP predict mortality, with troponins having the greatest impact (50%) [33]. Huang et al. also described that D-dimer, NT-proBNP, and Troponin-I were mainly positive in the most severe cases [49]. Qin et al.’s pioneering study identified that elevated troponins or NT-proBNP were highly predictive of all-cause mortality at 28 days [50]. Our study found similar outcomes regarding 30-day mortality. Based on these findings, biomarkers can serve as prognostic tools.

#### 4.1.8. Outcomes at 30-Day Follow-Up

The positivity of evaluated biomarkers is associated with disease severity and in-hospital mortality. However, after 30 days post-discharge, there was no statistically significant difference in mortality or readmission rates among the different groups (6.5%), though this was better than the 7.1% reported by Peiris et al. [51]. This result contrasts with Lionte et al., who found that NT-proBNP and Troponin I were predictors of 30-day mortality in an emergency hospital setting [52].

#### 4.1.9. Comparison between Subgroups

Among patients in group B (two abnormal or positive biomarkers), those with elevated troponins had the highest mortality and significant odds ratios (ORs), with ORs of 2.5 for the troponin + D-dimer group and 2.4 for the troponin + natriuretic peptide group. Current evidence is insufficient to relate COVID-19 mortality with concomitant positive biomarkers. Future research with a larger participant pool could provide a more accurate OR.

### 4.2. Strengths and Limitations

The strengths of this study include its multicenter design, encompassing various care centers across the continent and yielding a significant sample size (476 patients). All participants underwent thorough cardiac biomarker measurements, with a 30-day follow-up, collecting data from over 90% of participants.

However, the study’s prospective cohort design has intrinsic limitations, including the lack of randomization and susceptibility to selection biases due to the wide range of COVID-19 severity and variability in test protocols across institutions and regions. Additionally, the absence of quantitative follow-up of these biomarkers limits definitive conclusions regarding their evolution over time and their clinical utility.

## 5. Conclusions

In patients with COVID-19 requiring hospitalization in Latin America, the presence of at least one altered cardiovascular biomarker (Troponin, natriuretic peptide, and/or D-dimer) is associated with an increased number of cardiovascular complications, ICU admission, ICU respiratory/cardiovascular support, and in-hospital mortality. Thus, measuring these cardiovascular biomarkers during hospitalization could aid in patient stratification and the implementation of individualized treatment and follow-up strategies.

## Figures and Tables

**Table 1:** Demographics and comorbidities.

Variables	N	Number of Positive Biomarkers					*p*-Value ^2^
		Overall,	Group A,	Group B,	Group C,	Group D,	
		N = 476 ^1^	N = 118 ^1^	N = 190 ^1^	N = 139 ^1^	N = 29 ^1^	
Sociodemographic characteristics							
Age	476	64.0	69.0	65.5	58.0	49.0	<0.001
		(52.0–73.0)	(58.0–77.0)	(55.2–74.0)	(46.5–68.0)	(40.0–69.0)	
Gender	476						0.3
Female		151 (31.7%)	44 (37.3%)	60 (31.6%)	41 (29.5%)	6 (20.7%)	
Male		325 (68.3%)	74 (62.7%)	130 (68.4%)	98 (70.5%)	23 (79.3%)	
Comorbidities							
Hypertension	476	273 (57.4%)	79 (66.9%)	114 (60.0%)	69 (49.6%)	11 (37.9%)	0.005
Diabetes mellitus	476	162 (34.0%)	43 (36.4%)	71 (37.4%)	41 (29.5%)	7 (24.1%)	0.3
Dyslipidemia	476	79 (16.6%)	28 (23.7%)	39 (20.5%)	10 (7.2%)	2 (6.9%)	<0.001
Overweight/obesity	476	277 (58.2%)	60 (50.8%)	121 (63.7%)	81 (58.3%)	15 (51.7%)	0.14
Coronary heart disease	476						0.5
Clinical		29 (6.1%)	13 (11.0%)	9 (4.7%)	6 (4.3%)	1 (3.4%)	
Stent		18 (3.8%)	7 (5.9%)	7 (3.7%)	3 (2.2%)	1 (3.4%)	
Myocardial revascularization		5 (1.1%)	1 (0.8%)	2 (1.1%)	2 (1.4%)	0 (0.0%)	
Both		1 (0.2%)	0 (0.0%)	1 (0.5%)	0 (0.0%)	0 (0.0%)	
Heart failure	476	41 (8.6%)	21 (17.8%)	14 (7.4%)	4 (2.9%)	2 (6.9%)	<0.001
LVEF	36						0.3
LVEF < 40%		23 (63.9%)	12 (66.7%)	7 (53.8%)	3 (100.0%)	1 (50.0%)	
LVEF 40–50%		5 (13.9%)	4 (22.2%)	1 (7.7%)	0 (0.0%)	0 (0.0%)	
LVEF > 50%		8 (22.2%)	2 (11.1%)	5 (38.5%)	0 (0.0%)	1 (50.0%)	
Atrial fibrillation	476	25 (5.3%)	9 (7.6%)	10 (5.3%)	5 (3.6%)	1 (3.4%)	0.5
Stroke	476	10 (2.1%)	3 (2.5%)	6 (3.2%)	1 (0.7%)	0 (0.0%)	0.4

^1^ Median (IQR); *n* (%). ^2^ Kruskal-Wallis rank sum test; Pearson’s Chi-squared test. IQR: Interquartile range; LVEF: Left ventricular ejection fraction.

**Table 2:** Imaging Studies.

Variables	N	Number of Positive Biomarkers					*p*-Value ^2^
		Overall,	Group A,	Group B,	Group C,	Group D,	
		N = 476 ^1^	N = 118 ^1^	N = 190 ^1^	N = 139 ^1^	N = 29 ^1^	
Chest X-ray	476	455 (95.6%)	112 (94.9%)	180 (94.7%)	135 (97.1%)	28 (96.6%)	0.7
Pulmonary infiltrates	455						0.032
Unilateral		35 (7.7%)	4 (3.6%)	17 (9.4%)	10 (7.4%)	4 (14.3%)	
Bilateral		371 (81.5%)	99 (88.4%)	142 (78.9%)	113 (83.7%)	17 (60.7%)	
cardiomegaly	455	84 (18.5%)	36 (32.1%)	32 (17.8%)	15 (11.1%)	1 (3.6%)	<0.001
Lung congestion	455						<0.001
Unilateral		11 (2.4%)	3 (2.7%)	5 (2.8%)	3 (2.2%)	0 (0.0%)	
Bilateral		93 (20.4%)	41 (36.6%)	34 (18.9%)	18 (13.3%)	0 (0.0%)	
Pleural effusion	455						<0.001
Unilateral		25 (5.5%)	8 (7.1%)	14 (7.8%)	2 (1.5%)	1 (3.6%)	
Bilateral		27 (5.9%)	16 (14.3%)	8 (4.4%)	2 (1.5%)	1 (3.6%)	
Transthoracic echocardiogram	476	120 (25.2%)	44 (37.3%)	46 (24.2%)	26 (18.7%)	4 (13.8%)	0.002
Systolic function	119						0.3
Normal		78 (65.5%)	22 (50.0%)	32 (71.1%)	21 (80.8%)	3 (75.0%)	
Focal decrease		14 (11.8%)	8 (18.2%)	4 (8.9%)	1 (3.8%)	1 (25.0%)	
Global decrease		25 (21.0%)	13 (29.5%)	8 (17.8%)	4 (15.4%)	0 (0.0%)	
Right ventricular dysfunction	118	17 (14.4%)	14 (32.6%)	2 (4.4%)	1 (3.8%)	0 (0.0%)	<0.001

^1^ *n* (%); Median (IQR). ^2^ Pearson’s Chi-squared test; Kruskal-Wallis rank sum test.

**Table 3:** CV complications.

Variables	N	Number of Positive Biomarkers					*p*-Value ^2^
		Overall,	Group A,	Group B,	Group C,	Group D,	
		N = 476 ^1^	N = 118 ^1^	N = 190 ^1^	N = 139 ^1^	N = 29 ^1^	
Acute heart failure	476	84 (17.6%)	42 (35.6%)	35 (18.4%)	7 (5.0%)	0 (0.0%)	<0.001
Cardiac arrythmia	476	62 (13.0%)	27 (22.9%)	26 (13.7%)	7 (5.0%)	2 (6.9%)	<0.001
Miocarditis	476	13 (2.7%)	6 (5.1%)	5 (2.6%)	2 (1.4%)	0 (0.0%)	0.2
Pulmonary tromboembolism	476	22 (4.6%)	5 (4.2%)	8 (4.2%)	9 (6.5%)	0 (0.0%)	0.5
Other	476	75 (15.8%)	30 (25.4%)	27 (14.2%)	17 (12.2%)	1 (3.4%)	0.004

^1^ *n* (%); Median (IQR). ^2^ Pearson’s Chi-squared test; Kruskal-Wallis rank sum test.

**Table 4:** Treatment administered.

Variables	N	Number of Positive Biomarkers					*p*-Value ^2^
		Overall,	Group A,	Group B,	Group C,	Group D,	
		N = 476 ^1^	N = 118 ^1^	N = 190 ^1^	N = 139 ^1^	N = 29 ^1^	
Vasopressors	476	208 (43.7%)	59 (50.0%)	83 (43.7%)	60 (43.2%)	6 (20.7%)	0.043
Inotropes	476	83 (17.4%)	34 (28.8%)	32 (16.8%)	17 (12.2%)	0 (0.0%)	<0.001
Vasodilators	476	30 (6.3%)	14 (11.9%)	10 (5.3%)	6 (4.3%)	0 (0.0%)	0.024
IMV	476	240 (50.4%)	63 (53.4%)	99 (52.1%)	71 (51.1%)	7 (24.1%)	0.034
NIVM	476	87 (18.3%)	15 (12.7%)	43 (22.6%)	25 (18.0%)	4 (13.8%)	0.2

^1^ *n* (%). ^2^ Pearson’s Chi-squared test. IMV: Invasive mechanical ventilation; NIVM: Noninvasive mechanical ventilation.

**Table 5:** Outcomes during hospitalization.

Variables	N	Number of Positive Biomarkers					*p*-Value ^2^
		Overall,	Group A,	Group B,	Group C,	Group D,	
		N = 476 ^1^	N = 118 ^1^	N = 190 ^1^	N = 139 ^1^	N = 29 ^1^	
ICU admission	476	335 (70.4%)	87 (73.7%)	129 (67.9%)	103 (74.1%)	16 (55.2%)	0.15
Condition at discharge	476						<0.001
Alive		333 (70.0%)	59 (50.0%)	131 (68.9%)	114 (82.0%)	29 (100.0%)	
Dead		143 (30.0%)	59 (50.0%)	59 (31.1%)	25 (18.0%)	0 (0.0%)	
Type of death	143						0.066
Cardiovascular		42 (29.4%)	22 (37.3%)	17 (28.8%)	3 (12.0%)	0 (NA%)	
Not cardiovascular		101 (70.6%)	37 (62.7%)	42 (71.2%)	22 (88.0%)	0 (NA%)	

^1^ *n* (%). ^2^ Pearson’s Chi-squared test. ICU: Intensive care unit. NA: Not applicable.

**Table 6:** Outcomes after follow-up of 30-day.

Variables	N	Number of Positive Biomarkers	*p*-Value ^2^				
		Overall,	Group A,	Group B,	Group C,	Group D,	
		N = 333 ^1^	N = 59 ^1^	N = 131 ^1^	N = 114 ^1^	N = 29 ^1^	
Status	307						0.2
Alive		305 (99.3%)	50 (100.0%)	125 (99.2%)	102 (100.0%)	28 (96.6%)	
Dead		2 (0.7%)	0 (0.0%)	1 (0.8%)	0 (0.0%)	1 (3.4%)	
Rehospitalization	293	19 (6.5%)	5 (10.6%)	7 (5.8%)	6 (6.1%)	1 (3.7%)	0.6
Death cause	2						
Non cardiovascular		2 (100.0%)	0 (NA%)	1 (100.0%)	0 (NA%)	1 (100.0%)	

^1^ *n* (%); Median (IQR). ^2^ Pearson’s Chi-squared test; Kruskal–Wallis rank sum test.

**Table 7:** Relation of biomarkers with mortality.

		Overall, N = 190	Troponine + Natriuretic Peptide. N = 44	Troponine + D-Dimer. N = 25	D-Dimer + Natriuretic Peptide. N = 121
Condition at discharge	190				
Alive		131 (68.9%)	25 (56.9%)	14 (56.0%)	92 (76.0%)
Dead		59 (31.1%)	19 (43.2%)	11 (44.0%)	29 (24.0%)
	N	OR	CI 95%	*p*-value	
D dimer + natriuretic peptide	121	1			
Troponine + natriuretic peptide	44	2.4	1.16–4.99	0.018	
Troponine + D dimer	25	2.5	1.02–6.09	0.045	

## Data Availability

The data presented in this study are available in article.
